# A novel variant in the *COL4A3* gene: etiology of Alport syndrome type 2 in a 38-year-old male with suspected hereditary kidney disease

**DOI:** 10.1515/almed-2021-0058

**Published:** 2021-07-30

**Authors:** Paula Sienes Bailo, José Luis Bancalero Flores, Raquel Lahoz Alonso, María Santamaría González, Alex Gutiérrez Dalmau, Sara Álvarez de Andrés, Silvia Izquierdo Álvarez

**Affiliations:** Department of Clinical Biochemistry, Hospital Universitario Miguel Servet, Zaragoza, Spain; Department of Nephrology, Miguel Servet University Hospital, Zaragoza, Spain; NIMGenetics, Madrid, Spain

**Keywords:** Alport syndrome, *COL4A3*, familial glomerular hematuria

## Abstract

**Objectives:**

Patients with Alport syndrome develop progressive kidney function deterioration, sensorineural hearing loss, and ocular abnormalities. This condition is caused by mutations in *COL4A5* (X-linked inheritance), *COL4A3* and *COL4A4* (autosomal dominant or recessive inheritance), and encoding type IV collagen α3, α4, and α5, respectively. If left untreated, clinical symptoms progress from microscopic hematuria to proteinuria, progressive kidney failure, and end-stage kidney disease. At present, kidney transplantation is the only effective approach. Next-generation sequencing is the method of choice for the diagnosis of this condition.

**Case presentation:**

We report the case of a young man with chronic kidney disease who eventually underwent transplantation. Molecular testing made it possible to determine the etiology of his clinical symptoms and autosomal recessive Alport syndrome type 2. The patient was found to be a compound heterozygote for two missense variants (*trans* configuration) in the *COL4A3* gene: A likely pathogenic variant c.4981C>T (p.Arg1661Cys) in exon 52 inherited from the mother (described elsewhere), and another variant of uncertain significance, c.943G>A (p.Gly315Ser), in exon 17 inherited from the father that has not been previously reported in the literature or found in relevant databases.

**Conclusions:**

Following genetic confirmation, genetic counseling was provided to the patient and his direct relatives.

## Introduction

Alport syndrome (AS) is a rare disease characterized by the presence of structural abnormalities and dysfunction of glomerular basement membrane (GBM) and the basement membranes of other organs such as the eyes and ears. Patients with AS experience progressive deterioration of kidney function, with ultrastructural changes in the GBM, neurosensory hearing loss and ocular abnormalities [1, 2]. This syndrome is caused by mutations in *COL4A3*, *COL4A4* and *COL4A5*, and encoding type IV collagen α3, α4, and α5, respectively, present in the GBM and other basement membranes. Based on the mutated gene, three inheritance patterns have been identified: in 80% of cases, inheritance is X-linked (XLAS or AS type 1, MIM#301050), which is associated with mutations in *COL4A5*; whereas 15% is transmitted by autosomal recessive inheritance (ARAS or AS type 2, MIM#203780), and the remainder 5% is caused by autosomal dominant inheritance (ADAS or AS type 3, MIM#104200). Both, ARAS and ADAS are caused by mutations in *COL4A3* or *COL4A4* [3, 4]. In the case of ARAS, the lack of collagen α345(IV) expression in basement membranes is associated with a severe early-onset phenotype that affects men and women equally. The risk for end-stage renal disease (ESRD) in these patients is 100%, with the rate of progression and age at onset of extrarenal manifestations being genotype-dependent [5].

If untreated, clinical symptoms progress from microscopic hematuria to proteinuria, progressive kidney failure, and ESRD. Kidney transplantation is the only effective treatment currently available, although patients may benefit from early treatment with nephron-protective drugs including angiotensin-converting enzyme (ACE) inhibitors or angiotensin receptor blockers (ARBs), which delay progression to ESRD and improve life expectancy [6]. Therefore, early diagnosis of patients and relatives at risk is crucial. Genetic testing is the diagnostic method of choice, since it is less invasive than skin or kidney biopsy, with a diagnostic specificity of 95%. Sanger sequencing has long been the gold standard, although next-generation sequencing (NGS) emerges as a promising alternative [7, 8].

We report the case of a young man with chronic kidney disease who eventually underwent transplantation. Molecular testing made it possible to determine the etiology of his clinical symptoms, autosomal recessive AS type 2, with the identification of a novel variant of *COL4A3* not reported to date. Following genetic confirmation, genetic counseling was provided to the patient and his direct relatives.

## Case presentation

A 38 year-old male referred from the Nephrology unit in July 2019 for testing for alterations in collagen genes. The patient, with chronic kidney disease (CKD) attributed to basement membrane disease, underwent transplantation in 2014 and subsequently developed hearing loss. Without a relevant familial history, he was the only child of nonconsanguineous parents

At 3 years, he had low-grade fever and was diagnosed with asymptomatic urinary tract infection. At 4 years, he had two urinary tract infections with microhematuria and low-grade fever. At 5 years, the patient had recurrent tonsillitis and some episodes of dysfunctional voiding with microhematuria. From 5 years of age, the patient had recurrent macroscopic hematurias. Urine sediments contained granular casts and granular red blood cell casts with a high percentage of dysmorphic erythrocytes. Kidney biopsy confirmed a diagnosis of diffuse mesangial proliferative glomerulopathy. Recurrent hematurias and nephrotic-range proteinurias persisted, with normal renal function (Table 1).

**Table 1:** Pre/post-transplant variations in laboratory parameters of kidney function (2014).

	Reference interval	Pre-transplant										Post-transplant	
		2006	2007	2008	2009	2010	2011	2012	2013	2014	2014	Month 12	Year 6
												Nov/2015	Nov/2020
**Serum**													
Creatinine	0.67–1.17 mg/dL	1.00	1.30	1.21	1.19	1.52	1.57	1.58	3.74	4.79	6.76	2.10	2.18
Uric acid	3.5–7.2 mg/dL	7.8	8.9	8.3	7.6	5.7	5.4	4.4	8.7	8.1	10.9	7.50	4.81
eGFR (MDRD)	>60 mL/min/1.73 m^2^	–	–	71.93	72.79	54.49	52.13	51.41	18.90	14.11	9.48	36.33	35.01
Total proteins	6.6–8.3 g/dL	6.2	6.4	5.3	5.7	6.2	5.6	5.6	6.8	5.9	6.3	7.3	7.2
Albumin	3.5–5.2 g/dL	3.9	4.1	3.0	3.1	3.7	3.5	3.6	4.2	3.6	4.0	4.9	4.8
Potassium	3.5–5.1 mEq/L	4.5	4.8	4.9	4.2	5.2	5.1	5.2	6.9	5.6	7.4	4.4	4.0
Calcium	8.6–10 mg/dL	9.5	9.7	9.6	9.3	10.1	9.4	9.4	10.2	9.5	9.6	10.1	10.2
Phosphorus	2.5–4.50 mg/dL	3.8	3.5	3.7	4.2	4.2	4.7	4.0	4.1	5.3	4.2	3.30	3.50
Cholesterol	120–220 mg/dL	217	229	305	194	210	183	180	221	173	146	167	196
**Urine**													
Total proteins	<0.03 g/L	–	0.05	–	–	–	–	–	1.62	3.56	6.34	0.10	0.00
Total proteins (24 h)	<0.1 g/24 h	2.18	–	2.30	1.56	3.84	5.48	7.18	–	–	–	–	–
RBCs	0–5 per visual field	10–20	5–10	40–50	60–80	40–50	10–20	40–50	–	–	–	10–25	3–5

eGFR, estimated glomerular filtration rate; MDRD, Modification of Diet in Renal Disease.

Subsequent radiological studies showed signs of kidney disease, highlighting a megalic spleen with ectasia of the return vessels and an adenomatous/hyperplastic image. At kidney level, bilateral renal cortical hyperdensity was observed. At 29 years, despite treatment, the patient exhibited a progressive reduction of glomerular filtration and proteinuria. A repeat renal biopsy was performed including an ultrastructural study. Histological analysis showed thinning of the basement membrane of the glomeruli in the kidneys and images of splitting consistent with inherited kidney disease. The disease progressed into CKD (Table 1) confirmed by ultrasound (kidneys with preserved morphology and diminished corticomedullary differentiation). In a context of CKD progression and availability of a donor (his 65 year-old father, who did not show signs of kidney disease), the patient underwent kidney transplantation from a related living donor, with improvement of kidney function (Table 1).

In 2020, upon request of his nephrologist, targeted exome sequencing was performed in DNA extracted from an ethylenediaminetetraacetic acid (EDTA) peripheral blood sample by NGS (NIMGenetics, Madrid, Spain) to test for potential variants in a panel of 10 genes associated with Alport syndrome and other syndromes for differential diagnosis (*BSND, COL4A1, COL4A3, COL4A4, COL4A5, COL4A6, GATA3, MYH9, NPHS1,* and *NPHS2*). The variants identified were collated with the information contained in specific databases of other variants associated with a known phenotype (HGMD, ClinVar) and in databases of population frequencies (dbSNP, Genome Aggregation Database (gnomAD), 1000 Genome Project, NHLBI-ESP 6500 exomes). Additionally, the pathogenicity of the identified variants was estimated using eight prediction tools included in ANNOVAR (SIFT, PolyPhen2, LRT, MutationTaster, MutationAssessor, FATHMM, MetaSVM, and CONDEL) software and two additional (PROVEAN and Align GVGD) software packages for missense variants. To analyze gene conservation across species, sequence alignment was performed to compare nine amino acid sequences reported in collagen α3 (IV) chains of different species.

Genetic testing and segregation analysis revealed that the patient was compound heterozygote for two missense variants (*trans* configuration) in *COL4A3*: A likely pathogenic variant c.4981C>T (p.Arg1661Cys) in exon 52 inherited from the mother (described elsewhere), and another variant of uncertain significance, c.943G>A (p.Gly315Ser), in exon 17 inherited from the father that has not been previously reported in the literature or found in relevant databases (Table 2). The c.4981C>T (p.Arg1661Cys) variant was recorded in the HGMD database (CM014049) as associated with AS. Likewise, this variant had been previously included in the ClinVar database (Variation ID: 287915), with an interpretation conflict between pathogenic, likely pathogenic, and variant of uncertain significance. The c.943G>A (p.Gly315Ser) variant had not been recorded as associated with any specific phenotype.

**Table 2:** Mutations in *COL4A3* detected in the index case and his parents.

	Gene	Variant nomenclature	Exon	Zygosity	Effect	Variant classification	Inheritance	Phenotype
A	*COL4A3*	c.943G>A	17	Het	*missense*	VUS	AR	Alport syndrome type 2
		p.(Gly315Ser)						
	*COL4A3*	c.4981C>T	52	Het	*missense*	LPV	AR	Alport syndrome type 2
		p.(Arg1661Cys)						
B	*COL4A3*	c.943G>A	17	Het	*missense*	VUS	AR	Asymptomatic
		p.(Gly315Ser)						
C	*COL4A3*	c.4981C>T	52	Het	*missense*	LPV	AR	Asymptomatic
		p.(Arg1661Cys)						

Het, heterozygous; VUS, variant of unknown significance; LPV, likely pathogenic variant; AR, autosomal recessive. Genomic variants identified in: (A) next-generation sequencing of human exome in peripheral blood (clinical exoma) including 10 genes associated with Alport syndrome and syndromes included in differential diagnosis: *BSND, COL4A1, COL4A3, COL4A4, COL4A5, COL4A6, GATA3, MYH9, NPHS1,* and *NPHS2* (ExoNIM^®^ NIMGenetics). (B and C) Evaluation of the segregation pattern of the variants identified in the patient by Sanger sequencing: (B) in the father and (C) in the mother (NIMGenetics, Madrid, Spain).

## Discussion

We identified a novel variant in *COL4A3* in combination with a previously described compound heterozygous mutation. Mutations in the *COL4A3* gene are associated with AS type 3 and benign familial hematuria (MIM#141200) with autosomal dominant inheritance; and with AS type 2 with autosomal recessive inheritance. The clinical symptoms of the case reported (progressive severe proteinuria, hearing loss, and early-onset ESRD) was more consistent with AS type 2. This study demonstrates that molecular testing after the onset of symptoms is essential for early diagnosis and selection of the appropriate treatment.

**Figure 1:**
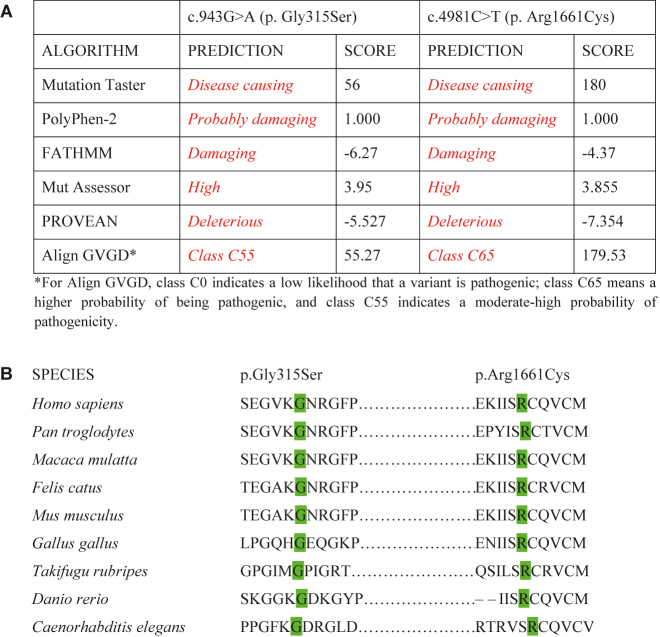
(A) Functional impact of the c.943G>A (p. Gly315Ser) and c.4981C>T (p. Arg1661Cys) variants and (B) interspecies conservation of the amino acids involved. (A) Results from several *in silico* prediction tools (MutationTaster, PolyPhen2, FATHMM, MutationAssessor, PROVEAN, and Align GVGD) in the pathogenicity analysis of the two mutations identified in the index case. These tools predict the potential impact of amino acid replacements on α3(IV) structure and function. (B) Alignment of partial amino acid sequence of α3(IV) in nine different species. The two positions (315 y 1661) are highly conserved (9/9) in evolution.

**Figure 2:**
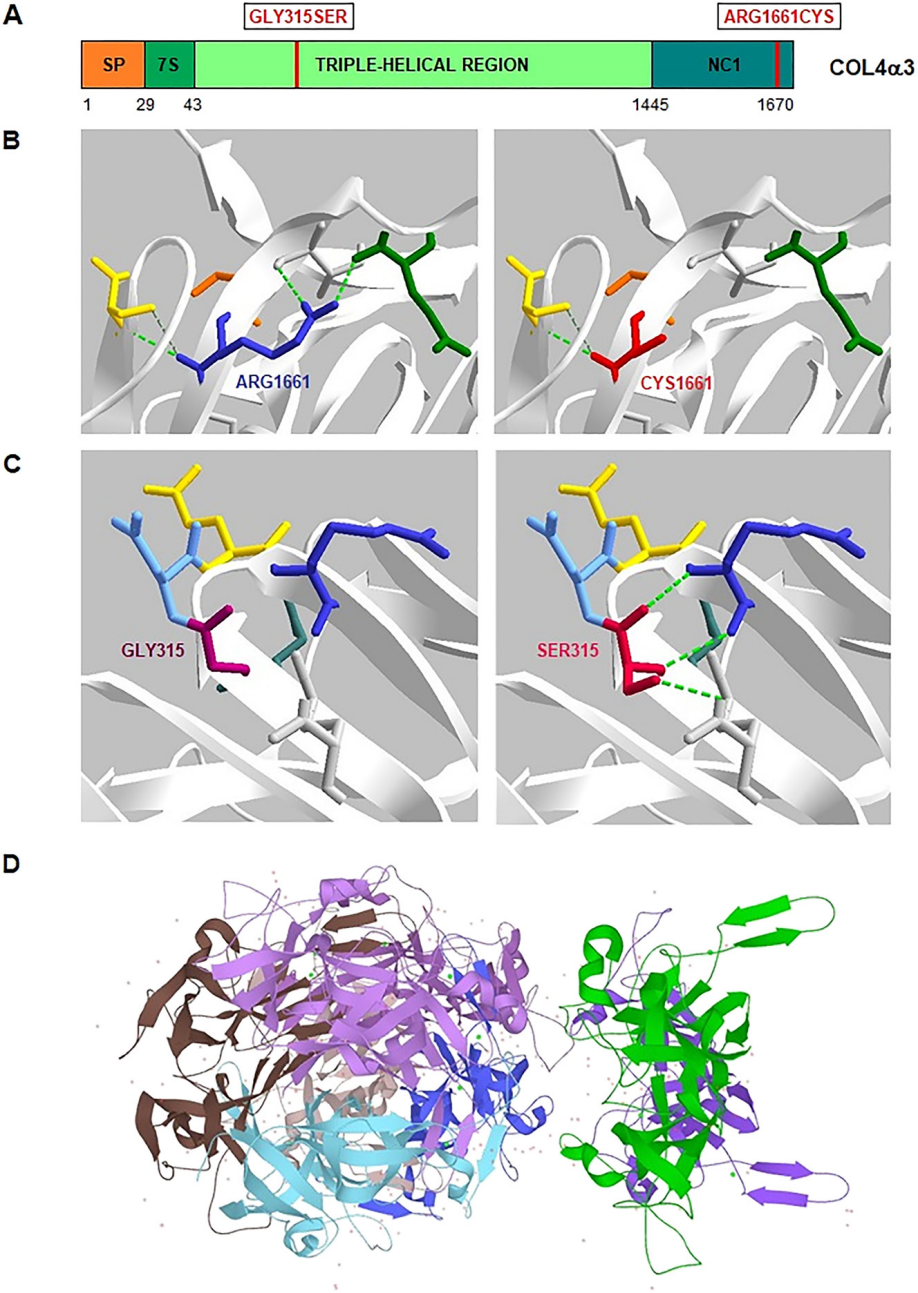
Collagen IV α3 chain, α3(IV), and 3D representation of the c.943G>A (p. Gly315Ser) and c.4981C>T (p. Arg1661Cys) variants. (A) Collagen α3 (IV) chains have a globular, cysteine-rich, C-terminal noncollagenous domain (NC1), frequent interruptions of GXY repeats in the central, long triple-helical domain that confer flexibility to the triple helix and a short triple helix domain (7S) in the N-terminal region. The graph indicates the location of the variants described: variant p.Gly315Ser is located in the central triple-helical domain, whereas variant p.Arg1661Cys is located in the NC1 domain. (B and C) 3D representation of variants in α3(IV) (PDB 5NB0, PDBviewer). In (B), replacement of the positively charged Arg1661 by the polar, smaller, uncharged Cys, alters hydrogen bonding patterns, hydrophobic and electrostatic interactions, and van der Waals contacts that are established with close amino acids eventually affecting the stability of the 3D structure and function of α3(IV). The same occurs in (C), replacement of Gly315, a polar uncharged amino acid, by a Ser, a larger, polar, uncharged amino acid, creates new hydrogen bonds that are absent in the native protein. (D) Overall 3D representation of the α3(IV) octamer (UniprotKB).

The c.4981C>T (p.Arg1661Cys) variant located in the C-terminal noncollagenous NC1 domain affects a highly conserved locus in the six collagen IV chains and alters the stability of the protomer and the NC1 domain structure, where cysteine is thought to form disparate disulfide bonds [9, 10] (Figures 1 and 2). This variant has been previously identified in other patients with ARAS, both in homozygosis [10] and in compound heterozygosis [10], [11], [12], diagnosed at a younger age (10–18 years), although an older age case (44 years) has been reported. As in our case, these patients exhibited glomerular basement membrane (GBM) thinning and nephrotic-range proteinuria. A patient also developed hearing loss and none had ocular abnormalities [12]. The c.943G>A (p.Gly315Ser) variant is presumed to have caused the clinical signs and symptoms of our patient, since it is segregated in the family, affects a highly conserved amino acid of the α3(IV) NC1 triple-helical domain and is suspected to exert highly negative effects and cause disease in most of the predictive models used (Figure 1). In addition, the presence of glycines arranged in a Gly-X-Y-Gly-X-Y pattern is important to the NC1 triple helix structure [9, 12] (Figure 2). A rapid course and early onset of ESRD (<20 years) in other carriers of the p.Arg1661Cys compound heterozygous variant could be secondary to a lower pathogenicity of the p.Gly315Ser variant as compared to Gly replacements in other positions (p.ej. p.Gly631Val o p.Gly830Asp) [10, 11].

The presence of heterozygous mutations in *COL4A3* is associated with a spectrum of phenotypes that ranges from the absence of symptoms to isolated, asymptomatic hematuria or even CKD, neurosensorial hearing loss and ocular abnormalities even within the same family [5, 9]. In this case, pretransplant genetic diagnosis would have contributed to better counseling about the risks associated with his father donation, since he was carrier of the variant of unknown significance. Of note, the father currently has preserved kidney function and is free of albuminuria or signs of progression into CKD. The mother, who is the carrier of the likely pathogenic variant, has also remained asymptomatic, although this variant has been reported to be associated with ESRD in heterozygous individuals [13].

Genetic testing of his offspring and other relatives at risk is indicated, as well as regular surveillance, with special attention being paid to the occurrence of hypertension, proteinuria, hematuria, or kidney failure. When heterozygous mutations are identified in *COL4A3*, initiation of therapy with ACE inhibitors or ARBs can be considered for the early prevention of progression into ESRD [14]. Hence, genetic testing emerges as essential in the diagnosis of kidney diseases such as AS, since accurate genetic diagnosis may lead to early diagnosis and treatment, prevention of rapid disease progression, appropriate selection of donor relatives and family planning [15].

## Lessons learned

– The presence of a novel variant concurrent to a previously described variant in *COL4A3* in compound heterozygosity is consistent with ARAS.
– The identification of the molecular etiology of kidney disease improves diagnostic accuracy and contributes to assessing the risk of recurrence in families, provide appropriate genetic counseling, and optimize treatments; thereby sparing the patient from ineffective therapies.
– Genetic testing in AS also helps exclude the presence of the disease in other members of the family, who may be candidate donors.
